# Efficacy of Psychoeducation and Cognitive Rehabilitation After Mild Traumatic Brain Injury for Preventing Post-concussional Syndrome in Individuals With High Risk of Poor Prognosis: A Randomized Clinical Trial

**DOI:** 10.3389/fneur.2019.00929

**Published:** 2019-09-04

**Authors:** Sophie Caplain, Gaelle Chenuc, Sophie Blancho, Sébastien Marque, Nozar Aghakhani

**Affiliations:** ^1^Psychopathology and Neuropsychology Laboratory, University Paris 8, Saint-Denis, France; ^2^Capionis Research, Bordeaux, France; ^3^Institut pour la Recherche sur la Moelle Epinière et l'Encéphale, Paris, France; ^4^Department of Neurosurgery, Bicêtre University Hospital, Paris, France

**Keywords:** mild traumatic brain injury, post-concussion syndrome, rehabilitation, quality of life, human

## Abstract

Unfavorable outcomes (UO) occur in 15–20% of patients with mild traumatic brain injury (mTBI). Early identification of patients at risk of UO is crucial for suitable management to be initiated, increasing the chances of full recovery. We previously developed a prognostic tool for early identification (8–21 days after the injury) of patients likely to develop UO. Patients whose initial risk factors indicate UO are at risk of developing post-concussion syndrome (PCS). In the present study, we examined the beneficial effects of early multidimensional management (MM) on prognosis. We used our prognostic tool to classify 221 mTBI patients into a UO (97) group or a favorable outcome (FO) group (124). We randomized the UO patients into two subgroups: a group that underwent MM (involving psychoeducation and cognitive rehabilitation) (34) and a control group with no specific treatment other than psychoeducation (46). At 6 months, these two groups were compared to assess the impact of MM. Among the followed-up patients initially classified as having FO (101), 95% had FO at 6 months and only five had PCS [as defined by the Diagnostic and Statistical Manual of Mental Disorders (DSM)-IV classification]. Among the followed-up MM patients, 94% did not have PCS 6 months after injury, whereas 52% of the control patients had PCS. The effect of MM on the recovery of patients at 6 months, once adjusted for the main confounding factors, was significant (*p* < 0.001). These results show that the initiation of MM after early identification of at-risk mTBI patients can considerably improve their outcomes.

**Clinical Trials Registration:** The study was registered at ClinicalTrials.gov (NCT03811626).

## Introduction

Mild traumatic brain injury (mTBI) accounts for 70–90% of brain injuries (1–3). Epidemiological studies show that it is difficult to accurately assess the number of cases of mTBI due to underdiagnosis (1, 4). Bazarian et al. (5) estimated that there are 600 cases of mTBI per 100 000 people in the united states, but only 100–300 mTBI patients per 100 000 people receive hospital-based care.

Symptoms reported immediately after injury tend to diminish over the following 10 days and are generally resolved by 3 months. However, in 15–25% of cases (6–14), problems persist, and may even worsen, at 3 months. Physical, emotional, and behavioral factors can be affected. Physical disorders include pain and fatigue. Sleep disorders are also common (14, 15). Persistent symptoms can affect patient outcomes (affecting all aspects of life) and increase public healthcare costs (16, 17).

## mTBI and Psychoeducation

In a recent systematic review of treatments offered to mTBI patients (18), the authors concluded that the most effective treatment was psychoeducation, which reduces long-term post-traumatic symptoms (9, 19). Providing explanations of the symptoms that can occur and of how they may progress can help patients to develop a less negative perception of mTBI. Such perceptions, even shortly after mTBI, seem to play an important role in the persistence of post-concussion syndrome (PCS) (20–22). Understanding the symptoms also helps patients to regain control over their disorder. Regular follow-up allows patients to contact appropriate medical personnel in the event of problems or questions that arise. Follow-up visits in the first or second week after injury are important, as they reduce social difficulties and post-traumatic symptoms at 6 months (23).

## mTBI and Cognitive Rehabilitation

Cognitive rehabilitation is intended to help injured patients to return to their pre-injury levels of performance. Its efficacy has been proven in other neurological disorders, but its efficacy in mTBI patients is still debated. Several studies, nonetheless, have shown positive results, notably in terms of working memory and attention (24, 25).

PCS is multidimensional and multifactorial (26). Considering that it is difficult to separate organic from psychological and environmental factors, several authors have put forward integrative models (20, 26–28). These models include the diathesis–stress paradigm proposed by Wood (28), which incorporates biological, psychological, and social factors, as well as cognitive, affective, behavioral, and environmental factors. This paradigm is based on the idea that an interaction between specific vulnerabilities and stressors triggers behavioral disorders in certain vulnerable individuals, and that vulnerability in some mTBI patients fuels post-traumatic symptoms. In PCS, all the features should be considered to be part of a global process in which each feature interacts with others, according to the different explanatory models of persistent PCS. This suggests that the management of mTBI patients should be holistic, while at the same time taking into account the individuality of each patient and of his or her own her pace of recovery.

Given how frequently mTBI occurs, it would be impractical to offer rehabilitation to all those affected. Moreover, as the outcome is favorable in most instances, it would also be inefficient. Ideally, management of mTBI ought to be performed in two stages: first, identification of patients with factors indicating a poor prognosis (with a risk of developing PCS) and, second, multidimensional management (MM) for the identified patients in order to reduce their handicaps and help them to return to a normal life. With this in mind, we previously developed a reliable (sensitive as well as specific) prognostic tool that enables early identification (within 15 days following injury) of at-risk patients (29). This prognostic tool is based on cognitive, somatic, mood, and quality of life (QoL) evaluation of each patient. The variables used for developing this prognostic tool are shown in Table 1.

**Table 1:** Variables used for developing our prognostic tool.

Complaints	Irritability, easily angered	
	Depressed, cries easily	
	Sensation of frustration, impatience	
	Memory loss and difficulty remembering	
	Difficulty concentrating	
	Slowed thinking	
QoL	VAS global QoL	
	Physical condition	
	Brain function	
	Feelings/emotions	
	Daily life	
	Social and personal life	
	Current and future situation	
Neuropsychological tests	Low-level treatments	TMT A T
		Stroop C
		Stroop W
	High-level treatments	TMT B-A
		Stroop W/C
		PASAT “correct answers” (CA)
		PASAT “telescoping errors” (TE)
		Verbal phonemic fluency “M”

Using this prognostic tool (29), in this study, we sought to establish whether an MM program (including psychoeducation and cognitive rehabilitation) effectively improves the prognosis of the patients identified as having a poor clinical and neuropsychological prognosis. The main aim was to assess the efficacy of early MM in mTBI patients deemed to be at risk of a poor outcome at 6 months. MM takes into account not only patients' cognitive features, but also their overall psychological state.

## Methods

The study was a multicenter, open, prospective, randomized study.

### Definition of mTBI

We used the diagnostic criteria of the American Congress of Rehabilitation Medicine (ACRM) Special Interest Group on Mild Traumatic Brain Injury: “a traumatically induced physiological disruption of brain function, as manifested by at least one of the following: (1) any period of loss of consciousness; (2) any loss of memory for events immediately before or after the accident; (3) any alteration in mental state at the time of the accident (e.g., feeling dazed, disoriented, or confused); and (4) focal neurological deficit(s) that may or may not be transient” and “where the severity of the injury does not exceed the following: loss of consciousness of 30 min or less; after 30 min, an initial Glasgow Coma Scale of 13–15; and post-traumatic amnesia not greater than 24 h” (30).

### Inclusion Criteria

Patients aged 18–65 who have mTBI, have healthcare coverage, can be followed-up for 6 months, and can understand French, reply in French, and cooperate.

### Exclusion Criteria

Intubation and/or ventilation and/or sedation upon arrival at hospital
Injury to the medulla, with signs of neurological impairment or multiple injuries (at least one of which is life-threatening)
Brain injury incurred during a suicide attempt
Psychiatric or psychological disorders that are debilitating and/or interfere with follow-up and/or evaluation
Psychoactive treatment ongoing at the time of injury
History of hospitalization in a specialized psychiatric setting and/or sick leave for psychological reasons
Neurological disorder
Substance dependence
Patient under guardianship or wardship.

### Assessment

At enrolment, all patients underwent an assessment at 8–21 days after injury that included a clinical examination and neuropsychological and psychological tests (described below). All patients underwent an end-of-study assessment at 6 months that was identical to the first assessment.

### Clinical Examination

History taking involved recording the patient's characteristics, the circumstances of the brain injury, any associated lesions, the signs of initial severity (Glasgow Coma Scale, neurological deficit, post-traumatic amnesia, and functional signs), computed tomography (CT) findings, if performed, management of patient between enrolment and day 8, and medication.

### Neuropsychological Evaluation

Psychologists/neuropsychologists assessed attention (vigilance or sustained, selective, or divided attention), working memory, reactive and spontaneous flexibility, and inhibition using the following neuropsychological tests:

### Cognitive Dimension

Digit span forward (Wechsler Memory Scale)
Digit span backward (Wechsler Memory Scale)
Mental control (MEM III, Wechsler Memory Scale)
Letter-number sequences (MEM III, Wechsler Memory Scale)
Trail Making Test, Parts A and B (31)
Stroop test (32)
Paced Auditory Serial Addition Test (PASAT) (33)
D2 Test of Attention (34)
Spontaneous flexibility was evaluated by categorical (Animal Fluency) and phonemic (letter “M”) verbal fluency, 1-min version (35)
Rey 15-item memory test (detection of simulators) (36).

### Psychological Evaluation

The following scales and questionnaires were used to evaluate QoL, mood, coping strategies, and symptoms:

10-point Visual Analog Scale for global QoL
Quality of Life after Brain Injury (QOLIBRI) questionnaire (37)
Hospital Anxiety Depression Scale (HADS A and D) (38).
State-Trait Anxiety Inventory A-trait scale (French version) (39)
Mini International Neuropsychiatric Interview (MINI): structured diagnostic interview corresponding to the criteria of the Diagnostic and Statistical Manual of Mental Disorders (DSM)-IV designed to detect mental illness (40).
Beck Depression Inventory
Brief COPE (coping strategies) (41)
Rivermead Post-Concussion Symptoms Questionnaire (RPCQ) (42)
Physical Evaluation
Epworth Sleepiness Scale (43)
Pichot Fatigue Scale (44)
10-point Visual Analog Scale for pain.

### Randomization

After the first assessment, and in accordance with the prognostic tool established in our earlier study (29), the patients were divided into two groups: favorable outcome (FO) and unfavorable outcome (UO) groups. The UO patients had initial risk factors that indicated risk of PCS. These UO patients were randomized into two subgroups: MM and control subgroups (described below). Simple (unrestricted) randomization using a computer random number generator was conducted. The final assessment at 6 months enabled comparison of these two subgroups and, hence, the impact of MM on outcomes.

### MM Group

Fourteen 1-h sessions were offered to patients with mTBI diagnosed as being at risk of UO and allocated to the MM group. The sessions began 1 month following injury and finished 6 months after injury. They were weekly during the first 2 months and fortnightly thereafter. The sessions involved psychoeducation (including regarding pain management), evaluation of mood disorders, and cognitive rehabilitation, as follows:

Sessions 1–3: Psychoeducation sessions on mTBI and its possible sequelae, and information on management, including pain management if necessary. Patients were referred for outpatient care, if required, so that they could receive suitable treatment (medication, physiotherapy, medical hypnosis, chiropractic treatment, osteopathy, etc.).

Sessions 4–9: Start of cognitive rehabilitation and management of mood disorders.

Sessions 10–13: Cognitive rehabilitation only.

Session 14: Last session of therapeutic management and 6-month neuropsychological and clinical evaluations.

Cognitive rehabilitation consisted of computer-based exercises (Paradigm® program), focused on addressing attention disorders, during the first half-hour. The exercises sequentially assessed the patient's attention levels, from the “intensity axis” (vigilance) to the “selectivity axis" (divided attention), as proposed by Van Zomeren and Brouwer. The exercises were made progressively more difficult and patients progressed to the next level only if they made no more than three mistakes.

Cognitive and behavioral management uses techniques designed to influence thought patterns as well as acquired behaviors and emotional characteristics. Information was collected in a semi-structured interview in the first session in order to identify difficulties encountered in daily life, and cognitive or emotional problems were recorded. The patient's expectations (and the degree of motivation regarding each expectation), perception of changes since the injury, locus of control, and intervention priorities were recorded, so as to identify what the rehabilitation specialist should work on to strengthen the patient's determination and consolidate the therapeutic alliance. In the first session, a questionnaire on the patient's perception of the accident was used to measure the mental impact of the accident on the patient and its intensity. At the beginning and end of each session, visual analog scales (from 0 to 10) were used to record the patient's level of pain (including headache), fatigue, anxiety, and depressive mood.

### Cognitive Rehabilitation Program

Standardized training was used, based on progressively harder exercises using a computer interface. The exercises and their order were identical for all patients and at each session. In contrast, the time spent on each level varied between patients, depending on their level and results. The exercises comprised parallel versions (runs) for a given level of difficulty, so the program could be run at a rate commensurate with the progress of each patient. The exercises were visual and auditory. The following aspects of attention were evaluated: vigilance, sustained attention, selective attention, and divided attention.

### Diagnosis of PCS

On day 180, patients were screened for post-concussion signs and PCS, according to DSM-IV criteria. The DSM-IV defines PCS using several criteria, but for research purposes rather than clinical use. This PCS definition was not included in the DSM-V, which, unfortunately, does not do justice to the current state of the medical literature when it comes to the data regarding the persistence of long-term symptoms following mTBI. The DSM-V states that, except in cases of severe TBI, the typical course is an improvement in neurocognitive, neurological, and psychiatric signs and symptoms. The DSM-V goes even further by stating that the neurocognitive symptoms following mTBI “tend to resolve within days to weeks after the injury with complete resolution typical by 3 months.” While technically true, this does not account for the 10–15% of mTBI patients whose signs and symptoms persist beyond 3 months and result in permanent neurocognitive, neurological, and psychiatric signs and symptoms. In its “Differential Diagnosis” section, the DSM-V suggests that if these symptoms persist, then the practitioner should consider alternative diagnoses such as physical or factitious disorders. In the DSM-V, PCS has been replaced by major neurocognitive disorders (defined as significant cognitive decline) or mild neurocognitive disorders (defined as modest cognitive decline). This distinction is not clear. For these reasons, it seems to us that the DSM-IV criteria for PCS are more reliable than the DSM-V criteria for analyzing persistent symptoms after mTBI. Our position is strengthened by the fact that several articles published after 2013 deal with persistent symptoms after mTBI using DSM-IV, and not DSM-V, criteria (45–47). Despite these differences, in practice, the major or mild neurocognitive disorders of the DSM-V cover PCS and, in our experience, patients diagnosed with PCS according to the DSM-IV all have major or mild neurocognitive disorders.

### Control Group

Patients with mTBI diagnosed as being at risk of UO and allocated to the control subgroup were seen at 1, 3, and 6 months after enrolment. They received information on the natural history of mTBI, and pain management, if necessary. As in the MM subgroup, the control patients were referred for outpatient care, if required, so they could receive suitable treatment (medication, physiotherapy, medical hypnosis, chiropractic treatment, osteopathy, etc.).

### Primary and Secondary Endpoints

The presence of PCS, using DSM-IV criteria, at 6 months was the primary endpoint of our study. A DSM-IV diagnosis of post-concussional disorder was made if criteria C, D, and E were satisfied in the structured interview and if at least one of the neuropsychological test scores was significantly different from the normal range. The secondary endpoints were cognitive performance, QoL, and symptoms recorded at 6 months after injury. The healthcare professional who conducted the MM sessions was different from the healthcare professional who conducted the final assessment, who was blinded to group allocation.

### Calculation of Sample Size

On the basis of our previous data (48, 49), we estimated a rate of loss to follow-up at 6 months of about 10% (we also attempted to reduce the rate by compensating patients and covering their cost of travel to the examination center). The same studies (48, 49) indicated that about 20% of mTBI patients had a poor prognosis at the outset. Based on the results of these previous studies (48, 49), for the sample size calculation, we selected QoL and symptoms as the principal measures of the efficacy of MM. We then applied appropriate statistical methods to calculate the sample size needed to avoid missed detection of an effect (type II error). The calculation was based on high power to prevent type II errors (power, 1-β = 0.9) and on a low significance level to prevent type I errors (significance level, α = 0.05). The calculation also considered the difference (Δ) between the FO and UO subgroups in terms of QoL, and the common standard deviation:

- Δ (mean difference between the two subgroups) = 3.53
- standard deviation = 4.33
- significance level, α = 0.05
- power, 1-β = 0.9.

Based on this information, the minimum sample size required to detect a significant difference between the subgroups was 30 patients in the control subgroup and 30 in the MM subgroup. Therefore, we had to recruit 350 patients in order to obtain 60 UO patients (randomized into the control and MM subgroups) that would be followed-up for 6 months.

Descriptive and inferential statistical analyses were applied to all data recorded at the evaluations soon after the injury and at 6 months. Intra- and inter-group comparisons between these two time points were conducted using parametric tests (Student's *t*-test and the chi square test) or non-parametric tests (Fisher's exact test if the theoretical number was <5 and the Wilcoxon signed-rank test if it was 5–30).

## Results

**Figure 1:**
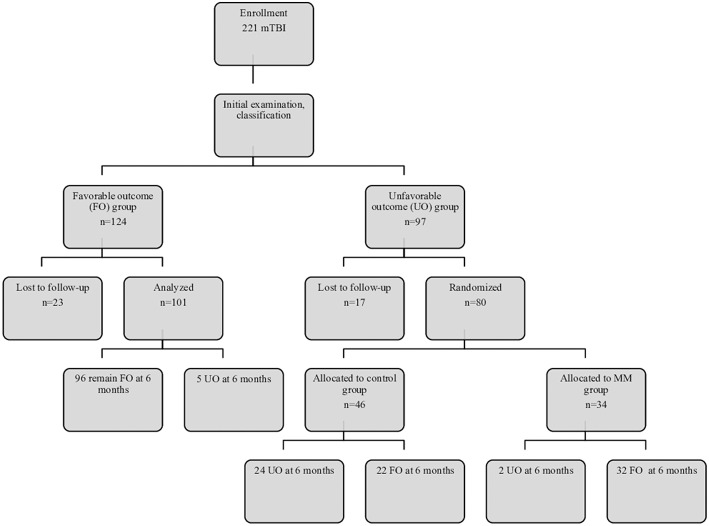
Flow diagram of subject recruitment, follow-up, and results. FO, favorable outcome; MM, multidimensional management; UO, unfavorable outcome.

During the year from January 2014 to January 2015, 124 (56.2%) of the 221 mTBI patients were classified as having FO, and 97 as having UO (43.8%). The groups were homogeneous in terms of age, sex, and socioeconomic status and educational level (Table 2), which reduced bias, as these factors influence the recovery time following mTBI (50, 51). The proportion of patients lost to follow-up was similar in the two groups (17/97 = 17.5% in the UO group and 23/124 = 18.5% in the FO group). Most cases of dropout were related to patients who did not wish to continue treatment that involved travel and time, which is partly explained by the long follow-up duration (Figure 1).

**Table 2:** Clinical summary of groups.

		**Favorable outcome (FO) (*n* = 101)**	**Unfavorable outcome (UO)**	
			**Control (*n* = 46)**	**MM (*n* = 34)**
Age, years	Mean	33.86	37.43	38.14
Gender, n (%)	Male	60 (59.4)	20 (43.47)	10 (29.41)
	Female	51 (51.49)	26 (56.21)	24 (70.59)
Level of education^*^, n (%)				
	1	4 (3.96)	5 (10.86)	3 (8.82)
	2	20 (19.6)	12 (26.08)	10 (29.41)
	3	18 (17.82)	10 (21.73)	3 (8.82)
	4	23 (22.77)	7 (15.21)	11 (32.35)
	5	36 (35.64)	12 (26.08)	7 (20.58)
Type of accident				
Attack		11 (10.89)	14 (30.43)	12 (35.29)
Fall		38 (37.62)	20 (43.47)	11 (32.35)
Other		10 (9.90)	4 (8.69)	3 (8.82)
Sporting accident		6 (5.94)	0	0
Road accident: car		3 (2.97)	3 (6.52)	3 (8.82)
Road accident: motorbike		16 (15.84)	1 (2.17)	2 (5.88)
Road accident: bike		7 (6.93)	4 (8.69)	2 (5.88)
Road accident: pedestrian		10 (9.90)	0	1 (2.94)
Work-related accident		17 (16.83)	4 (8.69)	9 (26.47)
GCS				
	14	4 (3.96)	1 (2.17)	1 (2.94)
	15	97 (96.04)	45 (97.83)	33 (97.06)
Initial loss of consciousness or post-traumatic amnesia		33 (32.67)	18 (39.13)	15 (44.11)

* GREFEX criteria: 1 ≤ lower school certificate; lower school certificate < 2 < higher school certificate; 3, higher school certificate; 4, diploma; 5, higher education.

*MM, multidimensional management*.

### Evaluation of the Efficacy of MM

Among the followed-up patients initially classified as having FO (*n* = 101), 96 (95%) had not developed PCS at 6 months, whereas five had PCS (according to DSM-IV criteria). In the MM subgroup, 94% did not have PCS at 6 months. In the control subgroup, 52% had PCS at 6 months (Figure 1). This shows that virtually all MM patients recovered within the 6 months following injury, whereas ~50% of control patients still had PCS. After adjusting for the main confounding factors, this positive effect of MM on patient recovery at 6 months remained significant (*p* < 0.001).

### Cessation and Resumption of Work

Among the patients initially classified as having FO, 38% stopped work for a mean of 9.7 days (1–30 days). Only six of these patients had not resumed work at the end of this study: four in the group that remained categorized as having FO (4.1%) and two in the group that became categorized as having UO (40%).

Among the control patients, 43% stopped work, compared with 48% of the MM patients. Of the control patients who stopped work, 85% resumed work (at the prior level of qualification in 80% of cases), while all MM patients resumed work (at the prior level of qualification, other than for one patient).

### Cognitive Outcomes

Compared with the control patients, there were no differences in the mean neuropsychological test scores at 6 months in the MM patients except for significant improvements in the PASAT subscore “no response” (*p* = 0.042) and in the phonemic fluency score “M” (*p* = 0.031). At 6 months, UO patients without PCS had worse mean cognitive scores than the FO patients. Control patients with PCS had worse mean cognitive scores than MM patients without PCS. Control patients without PCS had worse mean cognitive scores than the FO patients and the MM patients without PCS.

The neuropsychological test scores in the control patients were worse than in the MM and FO patients. However, the score was similar in the two UO subgroups (MM and control patients) in terms of speed of information processing evaluated using the mental control subtest. In the MM group, at 6 months there was an improvement in these scores, which rose to those of the FO group, whereas the scores did not change in the control patients without PCS by 6 months. Other functions improved between the start of the study and the 6-month follow-up in the MM group, but did not reach the levels in the FO group.

### QoL

There were significant differences in satisfaction with self-image, impairment related to physical problems, and QOLIBRI score between the control and MM patients, indicating a better QoL in the control patients. Overall, at 6 months, QoL was similar in the control patients with PCS and MM patients without PCS, while it was better for the control patients without PCS than for the MM patients without PCS.

### Symptoms

The mean number of symptoms in the MM subgroup at 6 months was significantly lower than in the control subgroup (*p* = 0.003). The MM subgroup had the same symptom profile as the FO group, albeit with more symptoms.

The mean degree of impairment in the MM subgroup was ≤1 (indicating “no symptoms” or “symptom without impairment,” except for fatigue). Although the MM patients without PCS at 6 months had more symptoms than the FO patients, on average, there was no impairment (≤1, symptom without impairment).

The mean intensity of symptoms in the control patients without PCS and the MM patients without PCS varied as a function of the type of symptoms but, on average, there was no impairment (≤1). The control patients with PCS had more symptoms with impairment (≤1) than the MM patients without PCS.

In summary, MM improved outcomes of UO patients by improving pathological parameters. It is important to point out that, at 6 months, the MM patients without PCS had on average more impairment than the patients classified as having FO at the outset. Control patients without PCS at 6 months had more impairment than MM patients without PCS.

## Discussion

The main aim of our study was to assess the neuropsychological and cognitive effects of an early MM program on the outcomes of mTBI patients who were identified early as being at risk of UO. In the MM subgroup, 94% had a favorable outcome, not developing PCS at 6 months, compared with only 48% of control patients. After adjusting for the main confounding factors, the effect of MM on the outcome at 6 months was significant (*p* < 0.001). Additionally, the results validate our prognostic tool, as 96 of the 101 followed-up patients (95%) initially classified as having FO did not develop PCS at 6 months, demonstrating the robustness of the classification. These results therefore support our main hypothesis that early identification of risk factors in mTBI patients can help avoid an unfavorable outcome (PCS) because MM can be implemented early after the injury. Almost all patients with early MM recovered.

The effects of MM seem relevant insofar as we noted cognitive improvements, with neuropsychological test scores that returned to normal. Our results show that the cognitive rehabilitation approach (centered on addressing attention disorders, involving an approach that sequentially addresses the intensity and selectivity of attention) enabled adaptation to the rhythm of each patient and more rapidly improved cognitive performance. On the other hand, the direct effects on QoL are less obvious, though improvement was noted in all aspects of life considered. Improvement of QoL in the MM subgroup was less than expected, as it did not reach the level in the FO group. MM limits the risk of persistent PCS, but seems insufficient to achieve a satisfactory level of QoL.

We have several explanations for the lower QOLIBRI score at 6 months in the MM patients without PCS compared with the control patients without PCS. One explanation is that, as a patient's QoL after brain injury encompasses all areas of daily life, a period longer than 6 months may be needed to regain full life satisfaction. Another explanation is that the MM program is time and energy consuming so the higher QoL in the control patients may be explained because they did not have to attend the MM program. Additionally, our initial prognostic tool had a sensitivity of 95.7% and a specificity of 77.6% (29), indicating that some patients could be incorrectly diagnosed as being at risk of UO when, in reality, they are not at risk and go on to report a high QoL at 6 months.

MM significantly reduced symptoms and the level of impairment. Symptoms, a criterion frequently used in the study of mTBI, are critical, given their subjective nature. Our study shows that from the early phase, the least injured patients, i.e., those in the FO group, had more than three symptoms, the most important of which were fatigue, sleep disorders, irritability, and concentration difficulties. These symptoms were more numerous in the two UO subgroups. At 6 months after injury, symptoms had decreased significantly in the FO group and the MM subgroup, as had the degree of impairment. However, symptoms remained quite numerous and were associated with a higher level of impairment in the control patients with PCS at 6 months. By virtue of their subjective character, symptoms are a fundamental guide for diagnosis. Whether or not they are correlated with objective indices, symptoms remain very informative regarding suffering experienced by mTBI patients and can guide early diagnosis of UO.

The fact that about 50% of the control group did not in fact have PCS at 6 months raises questions. This can partly be explained by the nature of the prognostic tool that we used in the early phase to categorize the patients. Our aim was to limit the risk of failing to identify some UO patients and it is possible that certain patients classified as being at risk were, in fact, at little risk of persistent PCS. Nonetheless, at 6 months, the control group did not completely achieve the level of the FO group and even exhibited worse cognitive performance than the MM patients without PCS. MM significantly improved the overall condition of patients at risk of UO, whereas spontaneous recovery of cognitive function would require more time without MM.

Commitment by patients to cognitive rehabilitation means that they can be treated using a therapeutic framework comprising a motivational process, which tends to increase the use of cognitive resources. Carefully listening to the patient and recognizing his or her physical and/or moral injury is fundamental to limiting the chronicity of problems, and it helps to free the patient of negative affect associated with the accident and of being labeled by others as a victim. Informing patients about mTBI and its sequelae seems to have been effective in some studies. However, in view of our results, this approach appears to be insufficient when the early injuries are substantial. Some 50% of control patients who were given the information still had PCS 6 months after injury. This is at odds with previous studies showing that psychoeducation alone is effective in some patients (9, 18, 20–22). The MM program seems to have added value in terms of improved recovery.

Early intervention to prevent persistent PCS appears to be beneficial. Both cognitive and behavioral therapies fit well in the MM program, as these therapies can be used to help patients adjust to their disorders, restoring their sense that they are in control of their body, mood, and activities of daily life. This type of preventive approach is a fundamental first step for any rehabilitative program and should continue to be used to disseminate knowledge about mTBI and its short- or long-term consequences, whether to the general public or to healthcare professionals.

## Study Limitations

One of the limitations of our study is the lack of accurate records of other treatments that patients received. Although we can confirm that the number of consultations outside our center was similar between the two groups, we cannot provide more precision on the nature of the consultations (i.e., which specialists were consulted).

Our results are encouraging, but it is still difficult to determine the extent to which the MM program has an effect specifically on cognitive recovery, given the entanglement of mood and cognitive and physical aspects. Such methodological limitations are highlighted when assessing MM, with unavoidable individual characteristics that are difficult to measure. More accurate evaluation of cognitive performance, particularly regarding attention, is needed for more precise cognitive rehabilitation. We chose to use conventional clinical assessment instruments that yield a fairly complete, but imprecise, cognitive profile. Finally, the cognitive rehabilitation materials we used require further refinement, notably by testing them on a greater number of individuals, so as to be able to establish standard values for each exercise and analyze the results in more detail.

## Conclusion

Our results confirm the importance of an early MM therapeutic approach for treating mTBI patients. First and foremost, the approach identifies patients at risk for persistent PCS who can then be offered therapeutic management in a timely fashion. This approach considerably improves the prognosis for these patients, which is an important issue for both public health as well as healthcare economics.

## Ethics Statement

The study was approved by the Ethics Committee of the Pitié-Salpêtrière University Hospital (ID RCB: 2012-A00015-38, Paris, France). Written informed consent was obtained from all participants.

## Author Contributions

SC, GC, SB, NA, and SM substantially contributed to the conception and design of the study, and to the analysis and interpretation of data. SC, SB, and NA contributed to the acquisition of data, involved in drafting the manuscript or revising it critically for important intellectual content, and in producing the approved final version for publication. All agreed to be accountable for all aspects of the work by ensuring that questions related to the accuracy or integrity of any part of the work are appropriately investigated and resolved.

### Conflict of Interest Statement

SM and GC were employed by Capionis Research. The remaining authors declare that the research was conducted in the absence of any commercial or financial relationships that could be construed as a potential conflict of interest.
